# Family‐centred care change during COVID‐19

**DOI:** 10.1111/nicc.12766

**Published:** 2022-03-02

**Authors:** Siriporn Vetcho, Marie Cooke, Helen Petsky, Amornrat Saito, Amanda J. Ullman

**Affiliations:** ^1^ School of Nursing and Midwifery Griffith University Brisbane; ^2^ Menzies Health Institute Queensland Griffith University Brisbane; ^3^ Faculty of Nursing Prince of Songkla University Songkhla; ^4^ Children's Health Queensland and Health Service, Centre of Children's Health Research Brisbane Australia; ^5^ School of Nursing, Midwifery and Social Work The University of Queensland St Lucia Australia

**Keywords:** family‐centred care, interdisciplinary professionals, neonatal intensive care unit, parents, Thailand

## Abstract

**Background:**

Family‐centred care (FCC) is an approach to promote family and health care provider partnership. This has been incorporated into neonatal intensive care units (NICUs) worldwide. However, FCC in low resource health settings, such as Thailand, is challenging and further impacted by coronavirus disease 2019 (COVID‐19).

**Aims:**

To evaluate FCC innovations to improve respect, collaboration and support in a Thai NICU.

**Study design:**

A quasi‐experimental study was conducted in an NICU in southern Thailand. Pre‐implementation was prior to COVID‐19, and parental and staff perceptions of FCC were measured via Perceptions of Family Centred Care‐Parent (PFCC‐P) and ‐Staff (PFCC‐S) survey. The FCC innovations were developed by stakeholders based on the COVID‐19 restrictions, pre‐survey results, parents' and clinicians' interviews and integrative review, then implemented via a flowchart. Post‐implementation evaluation was via repeated surveys. Comparisons were made pre‐and post‐implementation, with Mann–Whitney *U*‐test statistics for parents and Wilcoxon's Rank Sum for staff.

**Results:**

A total of 185 (85 pre; 100 post) parents and 20 (pre and post; paired group) health care professionals participated. Because of COVID‐19, many planned interventions were unfeasible, however, other innovations achieved (e.g., structured telephone updates, information booklet revision). There was an increase in parents' perception of respect ([median] 2.50–3.50), collaboration (2.33–3.33) and support (2.60–3.60) domains and overall (2.50–3.43; *p* < .001; 95% CI: 2.93–3.11). Interdisciplinary professionals' perception of FCC did not significantly change pre‐and post‐implementation/COVID‐19 pandemic for respect (3.00–2.92), collaboration (3.22–3.33), support (3.20–3.20) and overall (3.15–3.20; 95% CI: 3.10–3.25).

**Conclusion:**

Despite the challenges of COVID‐19 restricting NICU access, the provision of FCC was maintained and even improved.

**Relevance to clinical practice:**

Further research is necessary to develop FCC practice innovations associated with communication, across diverse health care systems and resources.

**What is known about the topic**

- In NICUs around the world, FCC is incorporated into clinical practice and widely used.
- Implementation of FCC in low‐resource health care settings is challenging due to complex and diverse political, social, cultural and economic characteristics.
- COVID‐19 has impacted care delivery for neonates and their families in NICUs, including visitation policies, developmental care and communication practices.

**What this paper adds**

- Implementation of practice innovations in NICU has improved parents and health care providers perceptions of the FCC elements of respect, collaboration and support.
- Despite COVID‐19, parents responded positively to the FCC innovations implemented into daily practice.
- Communication is necessary to work collaboratively with parents to promote partnerships in care during the social distancing and public safety of COVID‐19.

## 1 INTRODUCTION

Family‐centred care (FCC) promotes partnerships between parents (and other family members) and health care providers and encourages family participation in neonatal care.^1^ Galvin et al.^2^ and Hutchfield^3^ conceptually explored the critical elements of FCC from the perspective of both parents and staff and concluded that FCC consists of respect, collaboration and support. Practically, respect involves acknowledging individual family needs (e.g., spiritual, cultural), collaboration targets true partnerships in care planning and provision (e.g., care agreements), while support focusses on holistic assistance of family needs (e.g., help, sympathy).^2^, ^3^, ^4^ These three elements have become the critical aspects for measuring FCC perceptions.^4^

The neonatal intensive care units' (NICUs) complex and critical care environment is challenging for families.^5^ Neonates are separated from the family immediately after childbirth to provide life‐saving therapies. Consequently, it is difficult to establish a parental–neonate bond, which is essential for the physical, emotional and social well‐being of both the neonate and the parents.^6^, ^7^, ^8^, ^9^ Providing FCC during NICU admission can reduce stress and negative feelings.^10^ Components of FCC have been widely applied to clinical practice in NICUs worldwide,^11^, ^12^ resulting in benefits for the neonate, family and organization.^11^

Implementing FCC worldwide is challenging due to complex and diverse political, social, cultural and economic characteristics.^12^, ^13^ Differences in values and beliefs surrounding family, parenting, health and health‐care are culturally sensitive and impact how FCC is enacted, especially in neonatal critical care.^14^, ^15^, ^16^ In Thailand, the FCC concept has emerged into overarching models of care policy in neonatal care units^17^; however, paediatric nurses have indicated that they have difficulty incorporating FCC into daily practice.^18^ The role and responsibilities of Thai NICU nurses include administration of supportive and interventional therapies, such as medications and nutrition, and baby care routines focussed on growth and developmental processes.^19^ Thai nurse job descriptions follow the traditional hospital established system, with nurses having reduced authority in designing the services or provision of flexible care delivery.^20^ Additionally, due to staffing‐level nurses prioritizing routine physical care provision, responsive services for family needs are unmet.^21^

In Thailand, nurses' perceive FCC as a Western concept, with nurses' attitudes towards their roles, and a nursing shortage being obstacles to implementing FCC.^18^ Moreover, family needs, individuality and the Thai health care system further impact successful implementation of FCC.^18^, ^22^ Neonatal nurses have questioned whether the implementation of FCC in the NICU can contribute to changes in practice and organizations.^23^

COVID‐19 has impacted family interactions across health care, which has had the biggest impact on neonatal and paediatric intensive care units.^24^ Paediatric nurses in Italy identified that they provided a flexible service to maintain their FCC model; however, the impact this had on families and staff has not been explored.^24^ The international context of FCC in NICUs during COVID‐19 has not been explored, especially in low‐resource countries such as Thailand. During coronavirus disease 2019 (COVID‐19), parents' and interdisciplinary professionals' perceptions about the key elements of FCC in a Thai NICU are even more important.

### 1.1 Aims and objectives

This study aimed to evaluate whether practice innovations, implemented in a Thai NICU, facilitate FCC by improving parents' and interdisciplinary professionals' perceptions of respect, collaboration and support, including during COVID‐19.

## 2 METHODS

### 2.1 Design

The study was conducted using a quasi‐experimental design (pre, post) to evaluate the effects of FCC innovations developed based on current policies and practices in Thai NICU. The Ministry of Public Health of Thailand announced the first confirmed cases of COVID‐19 on 6 February 2020;^25^ however, changes to hospital policy regarding visitation were put into place from the 21 March 2020. The pre‐implementation period was prior to the COVID‐19 pandemic policy changes (February–20 March 2020), while the implementation (September–October 2020) and post‐implementation (November 2020–January 2021) periods were during COVID‐19.

### 2.2 Setting

The study was conducted in a 20‐bed, level IV NICU in a tertiary care hospital in southern Thailand with approximately 500 admissions per year from across southern Thailand (500 kilometres), approximately 32 nurses, two physicians (one professorial/senior specialist staff, one Resident) and one pharmacist.

### 2.3 Participants and sample

A purposive sampling approach was used to recruit parents and interdisciplinary professionals via daily screening of admission records. After seeking permission to approach parents from their health care provider, the researcher recruited participants (parents) during visiting hours. The interdisciplinary professionals were recruited through ward‐based advertising by internal mail using staff roster in‐service times. A sample size of 100 parents and 20 interdisciplinary professionals pre‐ and post‐implementation was feasible (i.e., availability of parents over three‐month periods; 57% of total staff).^26^ Parents and interdisciplinary professionals were required to satisfy the following inclusion criteria:

- Parents of neonates with an expected NICU stay of at least 72 h, who visited the NICU at least once. This included all gestational ages, however, infants with a life‐threatening or life‐limiting diagnosis and requiring palliative care were excluded from the study, due to the potential influence on parents' experience.
- Interdisciplinary professionals (nurses, physicians, pharmacist), with a permanent position providing care activities for at least 1 year in the NICU.
- Thai speaking and reading participants.

Neonates of participating parents were also included for descriptive purposes.

### 2.4 Ethical approvals

Ethical approvals were obtained from the hospital's and University Human Research Ethics committees (16th December 2019; 20th January 2020, respectively). Participants were provided with verbal and written information detailing the purpose of the study and right to withdraw consent. Confidentiality and anonymity of participants were maintained throughout the study by using unique participant numbers.^27^ Written consent was obtained.

### 2.5 FCC innovations

The FCC innovations were developed based on an integrative review,^28^ surveying parents' and interdisciplinary professionals' perception of FCC and interviewing nine parents and eight health professionals.^29^ Although a preliminary protocol for the intervention and implementation plan was considered within the NICU's current policies, COVID‐19 led to social distancing and public safety strategies regarding visitor restrictions in the NICU. Therefore, the FCC innovations were by necessity developed within the context of COVID‐19 visitor restrictions and staff workload challenges. The eight development working group participants were from key and high‐level stakeholders in the NICU, including the Head Nurse of NICU, the Sub‐Head Nurse of NICU, the In‐Charge Nurses, the bedside nurses and project leaders (including the external researcher, SV). Cross‐fertilization of ideas from the different speciality areas was incorporated,^30^ and the group developed a preliminary protocol for the FCC innovations and an implementation plan. The NICU medical professorial staff and the Deputy Director of Nursing of NICU reviewed the protocol before implementation.

The FCC innovations were considered within the current policies and practices of the NICU, including low‐scoring items from the pre‐intervention survey. In addition, the findings from the interviews (reported elsewhere)^20^ identified that the interdisciplinary professionals accepted the necessity of FCC for daily practice, but parents' participation in neonatal care was perceived as an obstruction to providing care.^29^ The FCC innovations included changes and updates to the detail within the parent information booklet including specific material related to COVID‐19; flexible visitation within the restrictions of COVID‐19 and structured communication checklists and documentation templates, which could facilitate FCC during the social distancing and public safety concerns. For its implementation, the flowchart of the innovations (Figure S1) was used to guide the practices with families in NICU. This flowchart simplified the process involved given the resource limits, timelines and workload challenges. The FCC innovations flowchart provided:

*Flexible visitation to daily updates (flexible hour) 1 h/day (over a flexible time period between 10 am–4 pm. (6 h) and restricted visitors to only parents, excluded during procedures and resuscitation.
*Telephone call (at least three times per week) to update newborn progress and treatment in NICU.
*Interdisciplinary family meeting for complex care situations.
- Information booklet, e‐booklet and paper‐based (revised) with the details for the COVID‐19 situation including NICU introduction: environment; staff; NICU policies; visiting management, important information and parental education during admission to NICU.

(*with structured communication checklist and documentation template)

To assist with implementation of these revised practices, in August 2020, there were theoretical, case study and practical training activities held by the development working group for the health care professional team (80% of team attended).

### 2.6 Data collection, context and instruments

Before COVID‐19, the NICU's visiting policy (the pre‐implementation) was 1 h, twice per day (10 am–12 pm, 1–2 pm.) and restricted visitors to only parents. However, during COVID‐19, there were no visitors allowed during the last quarter of March, and then restricted to 1 h/day during the pandemic (April 2020 onwards).

The parent and interdisciplinary professional surveys were conducted using the Perceptions of Family Centred Care–Parent (PFCC‐P) and Perceptions of Family Centred Care–Staff (PFCC‐S) instruments.^4^ The PFCC‐P and PFCC‐S were originally developed by Shields and Tanner^4^ in English and have been used in previous studies with further validation.^31^, ^32^, ^33^ Cronbach's alpha reliability coefficients of greater than 0.7 have previously been reported for the instruments across the three subscales.^32^, ^33^ The instruments consist of 20 items that are closely matched.^4^ The instruments are divided into three domains: respect, collaboration and support based on the items used by Hutchfield^3^ and Galvin et al.^2^ using a 4‐point Likert scale that have the advantage to obtain the perception of parents and staff. Respect includes six items around recognizing the family rights in the hospital. Collaboration reflects the partnership role of parents in caring for their neonate and comprises nine items. Support includes five items focussed on staff demonstrating support of the families' needs during the neonates' hospitalization. The response to each question is a Likert scale (never, sometimes, usually, and always), with scores ranging from 1 to 4, respectively.^31^ For each respondent, a median score was calculated for respect (average response for 9 items), support (average response for 6 items), collaboration (average response for 5 items) as well as overall.^31^

The PFCC‐P and PFCC‐S were translated from English into Thai, utilizing the guidelines for translation and cultural adaptation from the International Society for Pharmacoeconomics and Outcomes Research (ISPOR).^34^ Permission from the authors to use and translate the questionnaires was obtained. Cronbach's alpha coefficient of the PFCC‐P and PFCC‐S was 0.907 and 0.663, respectively.

In addition, demographic details were collected, including neonates', parents' and interdisciplinary professionals' characteristics, including the neonates' hospital records.

### 2.7 Data analysis

Demographic characteristics of parents, interdisciplinary professionals and neonates are reported using descriptive statistics, including frequencies (percent) and median (interquartile range [IQR]), relevant to data distribution and characteristics. Negatively worded items were reverse coded prior to calculation, as per previous uses of the FCC tool.^31^ The data were not normally distributed, so medians were used for each of the three sub‐scales. The statistical technique Mann–Whitney *U*‐test was used to analyse parents' perception on the items of the PFCC‐P in the pre‐and post‐implementation (unpaired groups). Wilcoxon's Rank Sum was used to compare the perceptions of the interdisciplinary professionals pre‐and post‐implementation using the PFCC‐S (paired groups).^35^, ^36^

## 3 FINDINGS

The participants consisted of 83 pairs of parents (i.e., mother and father of neonate participated) (35 pre; 48 post), which represented 102 neonates (50 pre; 52 post). There were 185 parents; 85 pre‐implementation and 100 post‐implementation. The pre‐implementation period stopped prior to 100 parents due to COVID‐19. The non‐equivalent sample in the group of parents was justified by the turnover of patients in the NICU. For the NICU health care team, 20 participated.

### 3.1 Parents

An equal number of males and females participated, as shown in Table 1. Most participants were aged between 21 and 30 years old (52%–60%), completed high school (39%–41%) and spent an average of 30 min–1 h travelling to the hospital.

**TABLE 1:** Demographic characteristics of parents and neonates admitted to NICU

Demographic	Pre‐ implementation		Post‐ implementation	
Parents	*N* = 85	%	*N* = 100	%
Gender (female)	42	49.4	51	51.0
Age group				
Less than 20 years old	1	1.2	8	8.0
21–30 years old	51	60.0	52	52.0
31–40 years old	31	36.5	29	29.0
More than 40	2	2.4	11	11
Educational level				
Elementary	23	27.1	31	31.0
High school	33	38.8	41	41.0
College	12	14.1	10	10.0
Bachelor's degree or above	17	20.0	18	18.0
Time to home				
Less than 30 min	28	32.9	34	34.0
30 min–1 h	22	25.9	40	40.0
More than 1 h	35	41.2	26	26.0
A previous child in NICU	5	5.9	5	5.0
Number of children under care				
One	49	57.6	63	63.0
Two	20	23.5	17	17.0
Three or more	16	18.9	20	20.0

Neonates	*N* = 50	%	*N* = 52	%
Gestational age (weeks) (median)	36.70 (26.2–41)		37.20 (26.2–41)	
Premature	25	50	24	46.2
Full term	25	50	28	53.8
Gender (male)	30	60	31	59.6
Delivery				
Vaginal delivery	35	70	47	90.4
Caesarean section	15	30	5	9.6
Primary diagnostic reason				
Prematurity	18	36	21	40.4
Infection	3	6	4	7.7
Congenital defect	3	6	4	7.7
Maternal risk: Hypertension	4	8		
Other	22	44	23	44.2
Admission weight (grams)				
Normal (>2500)	25	50	30	57.7
LBW (1500–2499)	11	22	11	21.2
VLBW (1000–1499)	8	16	9	17.3
ELBW (<1000)	5	12	2	3.8
Duration of incubator (days) (median)	7.50 (3–56)		6 (3–71)	
Mechanical ventilation				
Invasive (days) (median)	5 (1–28)		4 (1–66)	
Non‐invasive (days) (median)	3 (1–28)		2 (1–16)	
Length of stay (days) (median)	8 (3–56)		6 (3–71)	
Readmission in 30 days post discharge				
Never	50	100	49	94.2
Once	0	0	3	5.8

### 3.2 Neonates

There were notable demographic differences in neonates admitted to NICU between pre‐and post implementation. The most common diagnosis was prematurity, 36% (*n* = 18) pre implementation and 40% (*n* = 21) post implementation. The median length of stay was eight (pre) and six (post) days. All neonates pre implementation were not readmitted, while 5.8% post implementation were readmitted after NICU discharge (Table 1).

### 3.3 Interdisciplinary professionals

As a matched sample, the data are the same pre and post implementation. All participants were female and completed Bachelor's degree, mainly nurses (95%), aged between 31 and 40 years (40%), and had been working in NICU for 1–5 years (30%) (Table 2).

**TABLE 2:** Demographic characteristics of interdisciplinary professionals

Demographic	Pre and post implementation	
	*N* = 20	%
Age group		
21–30 years old	6	30
31–40 years old	8	40
41–50 years old	6	30
Time working with neonates (years)		
1–5	6	30
6–10	3	15
11–15	3	15
More than 15	8	40
Profession		
Physician	1	5
Nurse	19	95

Table 3 shows the scores of parents' and interdisciplinary professionals' perceptions of FCC pre and post implementation. The median scores of parents' perception in the post implementation significantly improved for respect (2.50–3.50), collaboration (2.33–3.33), support (2.60–3.60) and the overall scores (2.50–3.43; *p* < .001; 95% CI: 2.93–3.11). However, there was an absolute difference of at least 0.3 in the pre and post implementation scores for three subscales and overall scores, where 0.3 corresponds to 10% of the rating scale.

**TABLE 3:** Comparison of parents' and interdisciplinary professionals' perceptions of pre and post implementation–comparison by subscale and overall

Subscale	Parents median (IQR)		*p* value*	Interdisciplinary professional median (IQR)		*p* value**
	Pre implementation	Post implementation		Pre implementation	Post implementation	
Respect	2.50 (2–4)	3.50 (2.33–4)	.000	3.00 (2.33–3.67)	2.92 (2.33–3.50)	.166
Collaboration	2.33 (1.44–3.89)	3.33 (1.67–4)	.000	3.22 (2.56–3.67)	3.33 (2.67–3.89)	.467
Support	2.60 (1.2–4)	3.60 (2–4)	.000	3.20 (2.40–4)	3.20 (2.60–3.80)	.434
Overall	2.50 (1.7–3.8)	3.43 (2.3–4)	.000	3.15 (2.85–3.55)	3.20 (2.65–3.60)	.736

* Mann–Whitney test *p* ≤ .001 for all parent comparisons; Significant: *p* value <.05; 95% CI.

** Wilcoxon's Rank Sum test *p* > .05 for all interdisciplinary professionals comparisons; no significant: *p* value > .05; 95% CI.

The median scores of interdisciplinary professionals' perceptions of FCC pre and post implementation were not statistically significant different. However, after the implementation, there was a 2.75% improvement in the median score perception of the interdisciplinary professionals for the collaboration subscale (3.22–3.33). The respect subscale dropped slightly post implementation, and the support subscale was not different between pre and post implementation.

As would be expected from the median subscale scores, parents post implementation had significantly higher scores for all 20 PFCC‐P items than parents pre implementation (*p* < .05; 95% CI: 2.10–3.73). There was a difference of at least 0.3 between pre‐ and post‐implementation scores for 16 out of 20 items, including items: 2–5, 7, 9–14 and 16–20 (0.3 corresponds to 10% of the rating scale) (Table S1).

Regarding interdisciplinary professionals, there was an important difference (at least 10% of the rating scale) between pre and post implementation to demonstrate a more positive perception for item 2, “When parents come to the unit they feel welcome” and item 11 “Parents are taught what they need to know about their baby's care” (Table S1).

## 4 DISCUSSION

The key finding to emerge from this study was that parents positively perceived FCC improved, despite the restrictions associated with COVID‐19. Comparatively, the interdisciplinary professionals' total responses did not change, despite these same challenges.

The sample of this study is likely to be representative of their respective populations in Thai NICUs. For parents' demographic, education level and age, parenthood reflects Thailand's NICU parents' characteristics.^37^ Interdisciplinary professionals' characteristics of this study reflect the gender of the occupational groups in NICU and experience in NICU.^18^ Neonates had similar characteristics pre and post implementation, across most common diagnosis, admission weight, duration of incubation the median length of stay.

COVID‐19 has had profound influences on the provision of FCC delivery for neonates and families.^24^, ^38^ The restriction of socialization during COVID‐19 has significantly changed service delivery and interaction with families.^24^ Within the context of COVID‐19 and current policies and practices in the Thai NICU, innovations were conceived regarding the three key elements of FCC. The flowchart of innovations was used to change the organizational culture of providing FCC in NICU within this context. Furthermore, there were staff training activities for the interdisciplinary professionals around providing the FCC innovations within their daily practices. Providing NICU staff with education and support is recommended for delivering FCC that facilitates collaboration, respect and supports the family.^39^, ^40^

During COVID‐19, the social distancing and public safety concerns impacted the physical presence of parents and family members of the hospitalized neonate and affected how care was provided in the NICU.^41^ For this study, flexible visitation for daily updates, telephone updates and the interdisciplinary family meeting for complex care situations with structured communication checklists and documentation templates to facilitate meaningful communication between families and the interdisciplinary professionals successfully promoted partnerships and care collaborations despite challenges of COVID‐19 on visiting restrictions, workloads and staffing. Families' participation in decision‐making and neonatal care has been shown to promote a mutual partnership between families and health care professionals^36^ and improved parents' experience in the NICU.^42^ Furthermore, the information booklet with paper and e‐book versions facilitated communication to engage parents to work with the interdisciplinary team. The importance of FCC in NICU during a pandemic remains, with parents as partners in care, and requires adaptations to the clinical culture of the unit to ensure partnerships with families remain central to care practices. Maintaining the vital presence of parents in care with daily meaningful communication is crucial.^24^, ^38^

Providing FCC in NICU where there is restricted socialization from COVID‐19 requires effective communication and collaboration between families and health care providers.^43^ In this context, it is important to consider creating resources to engage a family with communication to facilitate and enhance partnership.^24^ Although technological devices have positively impacted families' experiences with the hospitalized neonate and parents' feelings of involvement in neonatal care during the admission when they cannot visit or remotely view their babies,^44^ the cost of telemedicine can be prohibitive. This is different from the Western countries in implementing FCC intervention, particularly in developing countries with limited resources or cultures.^13^

The FCC innovations were associated with a significant improvement of parents' perceptions for respect, collaboration and support subscales and in all items of the PFCC‐P during the challenging COVID‐19 pandemic. An important difference (at least 10% of the rating scale) was found for overall three subscales and 16 out of 20 items. In contrast, the collaboration subscale's interdisciplinary professionals' response improved slightly after the implementation (2.75%). However, a minor drop in the subscale of respect and support was not significantly different pre and post implementation. There was a change in the clinical culture on service delivery and interaction with families regarding social distancing and public safety during COVID‐19.^24^, ^41^ Similar results were found in a Brazilian study, which revealed significant improvement in parents' perception of FCC for all subscales, whilst a significant difference was only found in one item in both the respect and support domains for the health care team.^36^

The difference between parents' and interdisciplinary professionals' perception of post implementation for FCC could be interpreted that parents' experience of FCC may be more appreciable than interdisciplinary professionals, or perhaps the health care professionals' perception of their performance falls below their expectations of themselves. The interdisciplinary professionals might have provided answers that did not fully reflect what they were doing in the NICU. For example, a previous study reported that nurses perceived themselves implementing more FCC practices than they actually were providing.^45^ Moreover, interdisciplinary professionals might average their experience of implementing FCC as their general work responsibilities. It can be considered that the innovations as performed helped implement FCC strategies in the daily care routine, particularly during the challenging times of COVID‐19, reflected by increasing awareness of the health care providers surrounding embracing parental presence.

Although parents' personal experience in the hospital was limited given the visitation restriction (hours and visitors) of COVID‐19, parents perceived that they were involved in caring for their neonate with the health care professionals. This may have resulted in parents' appreciation of the FCC innovations, particularly in their interactions with interdisciplinary professionals, representing the reach of applying innovations focussed on three critical elements of FCC (respect, collaboration and support) in practice. In addition, it could be that perhaps the interdisciplinary professionals understood what parents expected in interactions with them. Communication and sharing information between parents and health care teams is the principal feature of FCC.^46^ Early and continuing communication has indicated positive involvement^47^ and improved the perception level of FCC both in parents and interdisciplinary professionals^48^ as well as improved parents' satisfaction with the NICU team.^42^

### 4.1 Limitation

The restriction of parental visitation during the social distancing and public safety concerns of COVID‐19 profoundly changed the clinical culture on service delivery and interaction with families. This is both a strength and limitation of these results. Although the restrictions on visitation challenged FCC in the NICU, COVID‐19 was an opportunity to reconsider care practices, using all available resources and innovations to enable new ways of providing FCC. This study explored the association of FCC innovations with outcomes, not causation. This study was implemented in a single‐site study; thus, the result of this study cannot be generalized outside of the setting. However, the broad, clear inclusion criteria facilitate replication in additional settings.

### 4.2 Implication for practice

Implementing innovations relating to the Thai health care context was essential to reinforce the effectiveness and sustainability of FCC, even prior to COVID‐19. In this crisis of COVID‐19, there is an opportunity to look at problems from the new perspective and create FCC innovations in NICUs that make it possible to maintain and even improve FCC implementation. These innovations can now be used to influence broader and more diverse strategies, including health care systems and resources. In addition, physical presence is not always possible, particularly in the social distancing context of COVID‐19. Therefore, it is imperative to develop family nursing interventions and provide sufficient resources to help health care providers work collaboratively with families to maintain a vital presence in daily communication in the NICU.

## 5 CONCLUSION

In this study, it was found that parents responded to the improvements in respect, collaboration and support from the implementation of FCC innovations, including information booklet revisions, flexible visitation timing, telephone call updates and interdisciplinary family meeting for complex care situations with structured communication checklists and documentation templates used in daily practice, despite the challenges of COVID‐19. FCC innovations in the NICU require targeted communication strategies to engage collaborative working between families and health care providers to support parents as partners in care in the setting. Further FCC innovations are recommended to expand and enrich communication whilst targeting health care settings and resources in different settings. It is vital that the needs of health care providers and families are well‐balanced throughout FCC innovation and implementation approaches.

## CONFLICT OF INTEREST

There are no conflicts of interest associated with this manuscript.

## AUTHOR CONTRIBUTIONS

All authors (**Siriporn Vetcho**, **Marie Cooke**, **Helen Petsky**, **Amornrat Saito**, and **Amanda J. Ullman**) cited on the title page have made substantial contributions to the article and agree with the content. This includes contributions in terms of (1) the conception and design of the study, acquisition of data or analysis or interpretation of data, (2) drafting the article or revising critically for important intellectual content and (3) final approval of the version to be submitted.

## ETHICS APPROVAL

Ethics approval was obtained from the Research Ethics Committee of Hatyai hospital (Protocol number 14/2563) and the Griffith University Human Research Ethics committee (GU Ref No: 2020/018).

## PARTICIPANTS CONSENT

Participants were provided with verbal and written information detailing the purpose of the study, right to withdraw consent, assurance of confidentiality, and chief investigator's contact details. Signed informed consent was received from all participants. The confidentiality and anonymity of all participants were maintained throughout the study by using a unique number for each participant and ensuring any identifying characteristics were removed.

## Supporting information


**Figure S1.** Flowchart for providing family‐centred care (FCC) innovations

**Table S1.** Comparison of parents' and interdisciplinary professionals' perceptions of pre‐and post‐implementation comparison by item
