# Supplementary figures and images for: Family‐centred care change during COVID‐19
Source: Nurs Crit Care. 2022 Mar 2;27(3):460–8. doi: 10.1111/nicc.12766 (PMC9115396; doi:10.1111/nicc.12766)

**Supporting information**

**
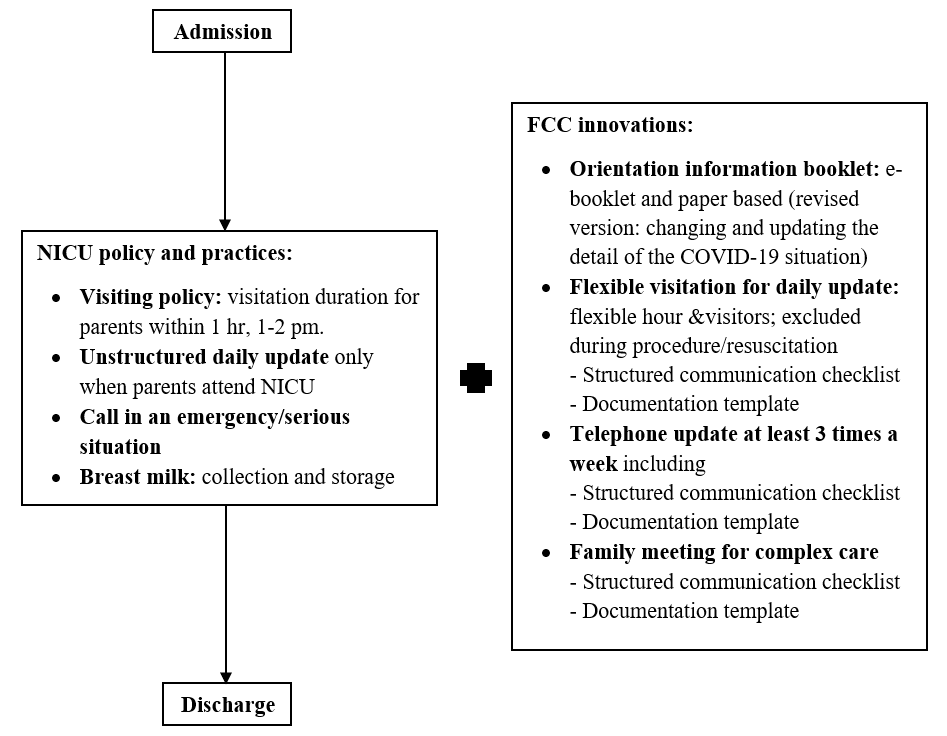
**

**Figure 1** Flowchart for providing FCC innovations

Supplement: Supplementary file 1 — Figure S1. Flowchart for providing family‐centred care (FCC) innovations [file NICC-27-460-s002.docx]
