# Supplementary material for: Family‐centred care change during COVID‐19
Source: Nurs Crit Care. 2022 Mar 2;27(3):460–8. doi: 10.1111/nicc.12766 (PMC9115396; doi:10.1111/nicc.12766)
Supplement: Supplementary file 2 — Table S1. Comparison of parents' and interdisciplinary professionals' perceptions of pre‐and post‐implementation comparison by item [file NICC-27-460-s001.docx]

**Supporting information**

**Table 1** Comparison of parents’ and interdisciplinary professionals’ perceptions of pre-and post-implementation comparison by item

| **Item^a^** | **Parents**  **Median (IQR)** | | **Z^*^** | **p-value** | **Interdisciplinary professional**  **Median (IQR)** | | **Z^**^** | **p-value** |
| --- | --- | --- | --- | --- | --- | --- | --- | --- |
|  | **Pre-implementation** | **Post-** **implementation** |  |  | **Pre-implementation** | **Post- implementation** |  |  |
| **Subscale: Respect (6 items)** | | | | |  |  |  |  |
| 1. When I come to the unit I feel welcome | 4 (2-4) | 4 (2-4) | -2.32 | .020 | 3.5 (2-4) | 4 (2-4) | -1.265 | .206 |
| 1. Other members of my family are welcome to attend with me in the unit | 1 (1-4) | 3 (1-4) | -7.05 | .000 | 2 (1-4) | 2 (1-4) | -1.508 | .132 |
| 1. I am able to be with my baby during procedures | 2 (1-4) | 4 (1-4) | -7.39 | .000 | 2 (1-3) | 2 (1-2) | -0.707 | .480 |
| 1. I am able to question recommendations about my baby treatment | 3 (2-4) | 4 (2-4) | -6.29 | .000 | 4 (3-4) | 4 (3-4) | -0.378 | .705 |
| 1. I feel like a visitor (rather than a parent) when I come to the unit | 3 (1-4) | 4 (1-4) | -3.60 | .000 | 3 (1-4) | 3 (3-4) | -0.832 | .405 |
| 1. My baby’s privacy and confidentiality are respected | 4 (1-4) | 4 (1-4) | -2.55 | .011 | 4 (3-4) | 4 (1-4) | -2.530 | .011 |
| **Subscale: Collaboration (9 items)** | | | | |  |  |  |  |
| 1. I feel prepared for discharge/referral to other community services after my baby’s discharge | 2 (1-4) | 3 (1-4) | -4.61 | .000 | 3 (2-4) | 3 (1-4) | -0.368 | .713 |
| 1. I am given honest information about my baby’s care | 4 (2-4) | 4 (2-4) | -2.39 | .017 | 4 (3-4) | 4 (3-4) | -0.000 | 1.000 |
| 1. I know who to call after I get home if I need help or reassurance | 2 (1-4) | 4 (1-4) | -5.33 | .000 | 3 (2-4) | 3 (2-4) | -1.000 | .317 |
| 1. When decisions are being made about my baby’s care the staff include me | 2 (1-4) | 4 (1-4) | -7.35 | .000 | 4 (3-4) | 4 (3-4) | -0.000 | 1.000 |
| 1. I am taught what I need to know about my baby’s care | 2 (1-4) | 4 (1-4) | -6.08 | .000 | 3.5 (3-4) | 4 (2-4) | -1.000 | .317 |
| 1. I know the name of the doctor in charge of my baby’s care | 1 (1-4) | 3 (1-4) | -6.42 | .000 | 3 (2-4) | 3 (2-4) | -0.258 | .796 |
| 1. I understand the written material that has been given to me | 3 (1-4) | 4 (1-4) | -5.69 | .000 | 3 (2-4) | 3 (3-4) | -0.000 | 1.000 |
| 1. My family is included in my baby’s care | 1 (1-4) | 4 (1-4) | -7.81 | .000 | 3 (1-4) | 3 (1-4) | -0.577 | .564 |
| 1. I feel overwhelmed by the information given to me about my baby | 4 (1-4) | 4 (1-4) | -2.42 | .015 | 3 (1-4) | 3 (2-4) | -1.327 | .185 |
| **Subscale: Support (5 items)** | | | | |  |  |  |  |
| 1. The staff are familiar with my baby’s individual needs | 3 (1-4) | 4 (1-4) | -4.99 | .000 | 3 (2-4) | 3 (3-4) | -1.232 | .218 |
| 1. The staff listen to my concerns | 3 (1-4) | 4 (1-4) | -5.31 | .000 | 4 (3-4) | 4 (3-4) | -0.378 | .705 |
| 1. I get to see the same staff | 2 (1-4) | 3 (1-4) | -4.85 | .000 | 3 (2-4) | 2 (2-4) | -1.807 | .071 |
| 1. The staff know who my support people are | 2 (1-4) | 4 (1-4) | -6.03 | .000 | 3 (2-4) | 3 (2-4) | -1.291 | .197 |
| 1. The staff understand what my family and I are going through | 3 (1-4) | 4 (1-4) | -6.94 | .000 | 3 (2-4) | 3 (2-4) | -0.237 | .813 |

Item^a^ labels are from the parent instrument (PFCC-P). The same items with minor rewording (mainly replacing “I” and “the staff” with “Parents” appeared on the staff instrument (PFCC-S)

*Mann-Whitney U test p≤ 0.001 for all parent comparisons; Significant: P-value < .05; 95%CI

**Wilcoxon’s Rank Sum test p> 0.05 for all interdisciplinary professionals comparisons; No Significant: P-value > .05; 95%
